# Causal Role of Thalamic Interneurons in Brain State Transitions: A Study Using a Neural Mass Model Implementing Synaptic Kinetics

**DOI:** 10.3389/fncom.2016.00115

**Published:** 2016-11-16

**Authors:** Basabdatta Sen Bhattacharya, Thomas P. Bond, Louise O'Hare, Daniel Turner, Simon J. Durrant

**Affiliations:** ^1^School of Engineering, University of LincolnLincoln, UK; ^2^School of Psychology, University of LincolnLincoln, UK

**Keywords:** thalamic interneurons, neural mass models, Lateral Geniculate Nucleus, kinetic modeling, synaptic kinetics, neurotransmitter concentration, alpha rhythms, neural oscillations

## Abstract

Experimental studies on the Lateral Geniculate Nucleus (LGN) of mammals and rodents show that the inhibitory interneurons (IN) receive around 47.1% of their afferents from the retinal spiking neurons, and constitute around 20–25% of the LGN cell population. However, there is a definite gap in knowledge about the role and impact of IN on thalamocortical dynamics in both experimental and model-based research. We use a neural mass computational model of the LGN with three neural populations viz. IN, thalamocortical relay (TCR), thalamic reticular nucleus (TRN), to study the causality of IN on LGN oscillations and state-transitions. The synaptic information transmission in the model is implemented with kinetic modeling, facilitating the linking of low-level cellular attributes with high-level population dynamics. The model is parameterized and tuned to simulate alpha (8–13 Hz) rhythm that is dominant in both Local Field Potential (LFP) of LGN and electroencephalogram (EEG) of visual cortex in an awake resting state with eyes closed. The results show that: First, the response of the TRN is suppressed in the presence of IN in the circuit; disconnecting the IN from the circuit effects a dramatic change in the model output, displaying high amplitude synchronous oscillations within the alpha band in both TCR and TRN. These observations conform to experimental reports implicating the IN as the primary inhibitory modulator of LGN dynamics in a cognitive state, and that reduced cognition is achieved by suppressing the TRN response. Second, the model validates steady state visually evoked potential response in humans corresponding to periodic input stimuli; however, when the IN is disconnected from the circuit, the output power spectra do not reflect the input frequency. This agrees with experimental reports underpinning the role of IN in efficient retino-geniculate information transmission. Third, a smooth transition from alpha to theta band is observed by progressive decrease of neurotransmitter concentrations in the synaptic clefts; however, the transition is abrupt with removal of the IN circuitry in the model. The results imply a role of IN toward maintaining homeostasis in the LGN by suppressing any instability that may arise due to anomalous synaptic attributes.

## 1. Introduction

The thalamic interneurons (IN) are believed to play a fundamental role in linking retinal sensory input to visual perception by modulating thalamocortical alpha rhythms (8–13 Hz) recorded through electroencephalogram (EEG) from the occipital cortex (Lörincz et al., 2009). Alpha rhythms are traditionally known to represent an idling state of the brain when a subject is awake but relaxed with eyes closed. Subsequently, it emerged that alpha rhythms play a key role in controlling perception and are of significance during an awake cognitive state. A transition of EEG from the alpha to the theta (4–7 Hz) band in an awake state is associated with several neurological disorders such as neurogenic pain, tinnitus, Parkinson's disease, and is termed as thalamocortical dysrhythmia (TCD) (Sarnthein et al., 2003; Hughes and Crunelli, 2005; Llinas et al., 2005); a similar symptom in Alzheimer's disease is termed as “slowing” (a decrease of dominant frequency) of the alpha rhythms. On the other hand, alpha to theta band transition in a quiet resting state is a marker of a change of brain state from wakefulness to drowsiness. In the visual pathway, local field potential (LFP) recordings of alpha and theta rhythms from the thalamic lateral geniculate nucleus (LGN) show a high correlation with EEG recorded simultaneously from the occipital cortex (Lopes da Silva et al., 1973; Hughes and Crunelli, 2006). In this regard, synchronous oscillatory patterns showing waxing-and-waning of amplitude within the alpha band is a well known hallmark of EEG and LFP in an awake and “resting” state (i.e., devoid of sensory input or mental task), and have been a matter of extensive research; their generation is attributed to the feed-forward and feed-back connectivity between cell populations of the Thalamocortical Relay (TCR: the main carriers of sensory information to the cortex) and the Thalamic Reticular Nucleus (TRN: a thin sheet of inhibitory cells surrounding the thalamus, receiving “copies” of communications between the TCR and visual cortex) (Steriade et al., 1990). Thus, computational models simulating thalamocortical dynamics have focused on the TRN as the primary inhibitory influence on the TCR, and thereby, on the cortex (Destexhe et al., 1996; Golomb et al., 1996; Stam et al., 1999; Robinson et al., 2004; Grimbert and Faugeras, 2006; Bhattacharya et al., 2011a; Wang et al., 2014); the inhibitory influence of IN is largely ignored. A similar gap is seen in experimental research investigating the functional impact of the IN cells on the thalamocortical oscillations (Crunelli et al., 2006; Halassa et al., 2014), exceptions being some early research in Crunelli et al. (1988) and Zhu et al. (1999a). This is in spite of the IN constituting around 20–25% of the total number of cells in almost all thalamic nuclei processing sensory information in mammals; around 47% of the synaptic afferents of the the IN are from the information carrying spiking neurons of the retina (Sherman, 2004; Jones, 2007). Moreover, the critical role of the IN in the visual signal processing by the LGN and information transmission in the retino-geniculo-cortical pathway is now well established (Dublin and Cleland, 1977; Wang et al., 2007; Babadi et al., 2010; Saalmann and Kastner, 2011; Wang et al., 2011a,b; Pressler and Regehr, 2013; Bastos et al., 2014; Hirsch et al., 2015); also, their physiology and spiking characteristics are now understood fairly well (Pape and McCormick, 1995; Zhu et al., 1999a,b; Cox et al., 2003). Thus, it is surprising that the importance of the causality of IN on brain rhythms is underestimated in experimental research, perhaps due to the lack of appropriate technology (Zhu et al., 1999a,b) that prevented proper recordings of the IN population dynamics. The emphasis on the role of IN in brain rhythms was revived only recently when Lörincz et al. (2008), while studying the waking state alpha rhythm and their response to cortico-thalamic inputs, report the distinct inhibitory effect of IN over TCR; this validates the findings in early research on IN cell dynamics (Crunelli et al., 1988) where the authors report the significant GABA-ergic influence of IN on the TCR oscillatory dynamics in the rat LGN. In addition, Lörincz et al. (2008) report a minimal role of the TRN on the TCR dynamics in the awake state. This is in agreement with a recent study using a computational model on the role of thalamic cells in the disappearance of alpha rhythms in sleep-wake transitions (Bond et al., 2014), where we have reported a dramatic effect of the presence of the IN cells on the TRN response; the research presented here builds on this prior work.

Neural mass computational models (NMM) (Marreiros et al., 2008; Moran et al., 2013) are often used to simulate brain rhythms recorded in LFP and EEG that are believed to be generated through dynamic interaction between networks of meso-scale (10^4^−10^7^ neurons) neuronal populations. These models were conceptualized in the works of Wilson and Cowan (1973) and Freeman (1975), and later popularized by the classic work on alpha rhythm by Lopes da Silva et al. (1974) and Zetterberg et al. (1978). Subsequently, this classic model was extended in Jansen and Rit (1995) and Suffczyński (2000), and used extensively in model-based research of neuro-psychiatric disorders (Wendling et al., 2002; David and Friston, 2003; Suffczyński et al., 2004; Modolo et al., 2013; Wang et al., 2013; Taylor et al., 2014). In previous works, we have proposed an enhancement to state-of-the-art neural mass models by replacing the “alpha function” (Rall, 1967) with kinetic models of Glutamatergic and GABA-ergic synapses (Bhattacharya et al., 2012; Bhattacharya, 2013). The motivation for these research has been to take a step forward in building computational tools that can complement experimental research in understanding the underlying cellular mechanisms of anomalous EEG signals in neurological and psychiatric disorders. The main inspiration for this approach has been the work by Destexhe (1994), where the authors state the following when discussing the future benefits of kinetic modeling of synaptic processes (p. 223): “A considerable amount of experimental data is available from measurements of the average activity of populations of brain cells: recordings of electroencephalogram, LFPs, magnetoencephalograms, optical recordings, magnetic resonance images, etc. It would be interesting to attempt to establish a relationship between such global measurements and dynamics at the molecular level.” Our work on NMMs embedded with synaptic kinetics showed a high sensitivity to the neurotransmitter concentration, forward and reverse rates of reactions during synaptic transmission, and the membrane conductance of the cell populations. Besides enabling the correlation of lower-level synaptic attributes to population-level dynamics in the model, the approach provided a 10-fold decrease in computational times compared to classic NMMs. The study presented here uses a neural mass computational model of the LGN implementing kinetics of α-amino-3-hydroxy-5-methyl-4-isoxazolepropionic acid (AMPA) and γ-amino-butyric acid (GABA) neuro-receptor mediated synapses, and is an extension of the model in Bhattacharya (2013).

For a long time, the only role of the thalamus was thought to be relaying sensory information to the cortex. The significant advances in our current understanding of other important functions of the thalamic circuitry, especially its integral role in cortical dynamics, is based upon some crucial and pioneering experimental investigations of the thalamic cells *in vitro* (McCormick and Prince, 1987; von Krosigk et al., 1993). Based on de-corticated (disconnected from the cortex) thalamic slices from the LGN of mammals and rodents, these works established an in-depth understanding of the intrinsic dynamics of the intra-thalamic cell populations (McCormick and Pape, 1990; Steriade et al., 1993; Pape and McCormick, 1995; Bal et al., 1995) and its fundamental role in cortico-cortical communication (Sherman and Guillery, 2002). In this work, our objective is to gain a thorough understanding of the synaptic dynamics of the IN afferents and efferents and their role in the LGN dynamics. In fact, a pioneering work identifying the significant effect of IN on the TCR cell dynamics constitutes of an *in vitro* experimental study on LGN slices from the rat brain (Crunelli et al., 1988). The model presented in this work endeavors to simulate the dynamics of a de-corticated LGN, similar to these early experimental studies.

The model output dynamics are observed under conditions of deletion of the IN synaptic pathways in the LGN circuit and corresponding to a set of “base” parameter values in the model as well as with parametric deviations. Our motivation toward such a model simulation set up is two-fold: to investigate the functional significance of the IN population on LGN oscillatory dynamics, considering that these cells are ignored in current model-based studies of thalamocortical oscillations; to investigate dynamical transitions in the model output that may arise with a dysfunction of the IN cell circuitry, which in turn may be caused by some abnormal pathological conditions in the network. The model input is a white noise simulating background spiking activity of the retinal neurons with eyes closed i.e., absence of visual input. Our results show that: First, if the IN population is disconnected from the network, both TCR and TRN population outputs display synchronous oscillations with waxing-and-waning of amplitude and power spectral peak frequency within the alpha band at ≈ 11 Hz: the hallmarks of EEG and LFP in an awake and resting state with eyes closed. Similar alpha rhythmic synchronous oscillations are also seen for reduced glutamatergic retinal input to the IN population (i.e., when IN is included in the network but receiving reduced visual input). Second, a “smooth” transition of the power spectra from the alpha to theta band is effected by a progressive decrease of neurotransmitter concentration in the synaptic clefts. In contrast, if the inhibitory effect of the IN is reduced by decreasing its efferent synaptic connectivity to the TCR below a certain threshold, a “dramatic” (as opposed to “smooth”) transition from alpha to theta band is observed in the model output when the levels of neurotransmitter concentration are lower than the set base value; the TCR and TRN enter a state of synchrony, showing waxing-and-waning amplitude patterns within the theta band. Third, increasing the ensemble leakage conductance of the IN cell population hyperpolarizes its average membrane potential, and both TCR and TRN time-series show synchronous oscillations in the limit-cycle mode and within the theta band. Thus, our results make a strong case for the IN as an integral part of the LGN circuitry in the awake cognitive state, maintaining an overall homeostasis in the system by preventing dramatic oscillatory state transitions in the TCR and the TRN, which appear when specific sets of synaptic attributes linked to the IN are altered.

As a case study, the model is tested for simulating Steady State Visually Evoked Potentials (SSVEP)—brain signals corresponding to flickering visual input at a constant or slow-varying frequency that can be observed through EEG. The frequency of the SSVEP signal is easy to control through the visual input, thus making it popular as a tool to study both lower-level and higher-level vision (see Norcia et al., 2015 for a review). The primary advantage of SSVEP is that the response frequency follow that of the input stimulus, making it possible to distinguish between multiple stimuli at different frequencies. It is therefore not surprising that SSVEP is growing in popularity for applications in clinical neuroscience aimed at understanding brain diseases (see Vialatte et al., 2010 for a review). In addition, Brain-Computer Interface (BCI) research have also used SSVEP for advancing current state-of-the-art applications (Guger et al., 2012). Thus, it seems appropriate that a model simulating LGN dynamics be tested for SSVEP response corresponding to a cognitive brain state. A visual stimulus input to the model is simulated by superimposing the (above-mentioned) white random noise with periodic impulse trains at a single frequency within the range 5–50 Hz. For all frequencies in this range, the fundamental frequency of the model output reflects the frequency of the input impulse train and has distinct harmonic components, thus agreeing with existing literature on SSVEP characteristics (Vialatte et al., 2010; Norcia et al., 2015). Furthermore, the IN and TCR cells in the model show similar power spectra characteristics and their time-series are in-phase. However, the TRN time-series is in anti-phase (approximately) with TCR. There is a distinct change in the model behavior when the IN is disconnected from the circuit: the TRN and TCR time-series are now in-phase (approximately) and the resonant frequency of the circuit dominates the power spectra, thus implying a reduced effect of the periodic stimulus input.

The model structure, parameterization, and simulation methods are discussed in Section 2. Results are presented in Section 3 along with discussions on their implications and model-based predictions. We conclude in Section 4 and mention future directions that will build on this work.

## 2. Materials and methods

**Figure 1:**
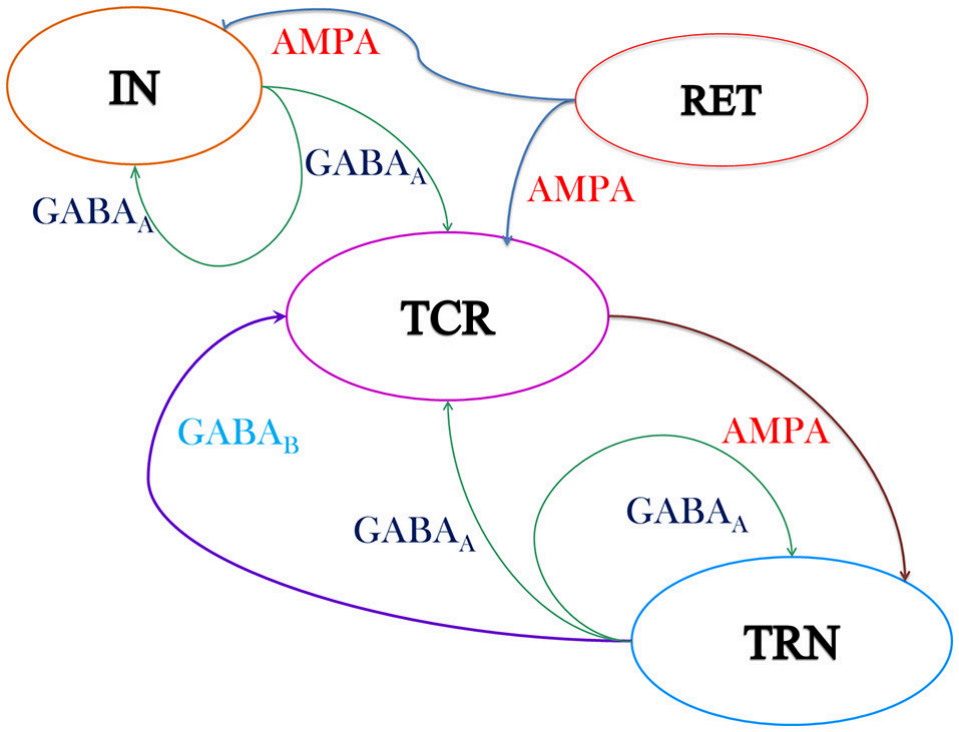
**Schematic of the neural mass model of the thalamic Lateral Geniculate Nucleus (LGN) consisting of three cell populations viz. the Thalamocortical Relay (TCR), the Interneurons (IN) and the Thalamic Reticular Nucleus (TRN)**. The excitatory synapses are modeled with AMPA kinetics while the inhibitory synapses are modeled with GABA_*A*_ and GABA_*B*_ kinetics. The output of the TCR population is taken as the model output, while the model input is assumed to be mean activity of the retinal spiking neurons (RET). The model “base” (reference) parameter values are tuned for dominant alpha rhythms in the model output and are given in Tables 1, 2. The intra-thalamic population connectivities are based on experimental findings (Sherman and Guillery, 2001) and are discussed in Section 2.2. Both AMPA and GABA_*A*_ are ionotropic (fast) synapses and are modeled with one first order differential equation (Equation 2). The GABA_*B*_ is a metabotropic (slow) synapse and is modeled with two sets of first order differential equations (Equations 3–5).

A schematic of the model used in this work is shown in Figure 1. The synaptic layout of the model is based on the intra-thalamic connectivity presented in Sherman (2006) and Sherman (2007), which in turn are informed by experimental data obtained from the LGN of mammals and rodents and are as reported in Van Horn et al. (2000), Sherman and Guillery (2001) and Jones (2007). In addition, recurrent feedback in both IN and TRN (Huntsman et al., 1996) populations are observed in the experimental studies and are included in the model.

The output of the TCR cells form the main source of sensory information to the visual cortex. Furthermore, in simultaneous studies on LGN and cortical outputs, the LFP from the TCR cells are observed to have a high coherence with EEG from the occipital scalp electrode (Lopes da Silva et al., 1974; Hughes and Crunelli, 2006; Bastos et al., 2014). Thus, in this model of the LGN, the time-series of the TCR cells is considered as the output and is hereafter referred to as the “model output.” Input to the model represent the mean activity of the retinal spiking neurons (the afferent population to the LGN) when the brain is in an awake state with eyes closed.

The retinal spiking neurons (ganglion cells) make excitatory (glutamatergic) synapses with the TCR and IN population of the LGN that are mediated by both fast ionotropic (iGluR) and slow metabotropic (mGluR) glutamate neuro-receptors (Cox et al., 2003; Wang et al., 2011b). However, Pape and McCormick (1995) report that in the presence of iGluR, mGluR have minimal effect on the IN membrane potential and their synaptic activities. This observation is subsequently confirmed by Govindaiah and Cox (2006). Hence, this pathway is often ignored in synaptic circuits of the LGN (Sherman, 2006). We follow the latter work, and consider only the ionotropic AMPA (iGluR) neuro-receptors mediated synaptic efferent pathways from the retina to both IN and TCR populations. The IN cells make feed-forward inhibitory (GABA-ergic) synapses on the TCR cells mediated by the ionotropic GABA_*A*_ neuro-receptors. The TRN cells receive excitatory synapses from the TCR population mediated by ionotropic AMPA neuro-receptors, and send inhibitory feedback to the TCR cells mediated by both ionotropic GABA_*A*_ and the metabotropic GABA_*B*_ neuro-receptors. In addition, the cells in both TRN and IN make feedback connections on their respective self populations, mediated by the ionotropic GABA_*A*_ neuro-receptors.

It is worth mentioning here that all thalamic nuclei that process sensory information are reported as having a similar architecture to that of the LGN (Sherman and Guillery, 2001; Saalmann and Kastner, 2011). Thus, the model in Figure 1 can also be used for simulating the thalamic dynamics corresponding to other sensory pathways. The mathematical framework for the model is mentioned in Section 2.1; model parameterization is discussed in Section 2.2; simulation methodologies are mentioned in Section 2.3. The reference values, referred to in this work as “base values,” of the model parameters are mentioned in Tables 1, 2.

Table 1**(A) Data for the forward (α) and reverse (β) rates of synaptic transmission is according to the range mentioned in Golomb et al. (1996) and Destexhe et al. (1994)**.

**Table:** 

**(A) NEUROTRANSMISSION PARAMETERS**		
**Parameters**	**Value**	**Synaptic pathway**
α ((mM) ^−1^. (s)^−1^)	1000	AMPA, GABA_*A*_
β ((s)^−1^)	50	AMPA
	40	GABA_*A*_
α_1_, α_2_ ((mM) ^−1^. (s)^−1^)	10, 15	GABA_*B*_
β_1_, β_2_ ((s)^−1^)	25, 5	
$g_{max}^{syn}$ (μS/cm^2^)	300	AMPA (RET to TCR)
	100	AMPA (RET to IN)
		(TCR to TRN)
	100	GABA_*A*_
	60	GABA_*B*_
$E_{rev}^{syn}$ (mV)	0	AMPA
	−85	GABA_*A*_ (TRN/IN to TCR)
	−75	GABA_*A*_ (TRN (IN) to TRN (IN))
	−100	GABA_*B*_ (TRN to TCR)
K_*d*_	100	GABA_*B*_
*n*	4

**Table:** 

**(B) CELL MEMBRANE PARAMETERS**		
	**RET**	**TCR**	**IN**	**TRN**
g^*leak*^ (μS/cm^2^)	X	10	10	10
E^*leak*^ (mV)	X	−55	−72.5	−72.5
V_*rest*_ (mV)	−65	−65	−75	−85

Note that the units used in our model are at a different time scale (sec^-1^), and thus absolute figures are different from these references. The data for maximal synaptic conductance $g_{max}^{syn}$ is in the range mentioned in (Wang et al., 1995; Golomb et al., 1996); note that the unit for this parameter in our model is μS/cm^2^. Data for E_rev_ is as in (Wang et al., 1995; Golomb et al., 1996). Specific data relating to the thalamic IN synapses are not mentioned in any of these sources, and are set as similar to those of TRN in this work. The ‘RET’ in the parameter superscripts refer to the retina as the source of input to the model. (B) The leakage current in the model cell populations are assumed to be due to Potassium (K) mainly. Thus, the leakage conductance and reverse potentials parameters in the model are in the range mentioned in (Wang et al., 1995; Golomb et al., 1996). The resting state membrane potential for TCR and TRN are as in (Wang et al., 1995), and that for IN is set arbitrarily to a depolarised (hyperpolarised) value with respect to the TRN (TCR). The resting membrane potential for the retinal spiking neurons (RET) is set at −65 mV, and their efferent signal to the TCR is simulated by a white random noise with mean −65 mV and standard deviation 2 mV^2^. Thus, there is no ODE corresponding to the RET in the model, and its leak conductance and leak reversal potentials are indicated with a ‘does not matter’ symbol ‘X’.

**Table 2:** **Base values of the synaptic connectivity parameters ***C***_***uvw***_ in Equation (6) are derived from experimental data on LGN of mammals and rodents (Van Horn et al., 2000; Sherman and Guillery, 2001; Jones, 2007) (see Section 2.2 for a brief overview)**.

**Efferents →**	**TCR**	**IN**	**TRN**		**Retinal**
**Afferents ↓**			**GABA_*A*_**	**GABA_*B*_**	
TCR	X	C_*tii*_	${\text{C}}_{tni}^{a}$	${\text{C}}_{tni}^{b}$	C_*tre*_
		$\frac{1}{2}$ of 30.9	$\frac{3}{8}$ of 30.9	$\frac{1}{8}$ of 30.9	7.1
IN	X	C_*isi*_	X	X	C_*ire*_
		23.6			47.4
TRN	C_*nte*_	X	C_*nsi*_	X	X
	35		20		

Each parameter value is a normalized figure that represents the percentage of the synaptic contacts made on the post-synaptic cell population u by the pre-synaptic cell population v, and w represents the sign of the synapse i.e., excitatory (e) or inhibitory (i). The afferent populations are represented by the letters t for TCR, n for TRN, i for IN and r for retina. For synaptic contacts by a cell population on itself, v is represented by s, which stands for a connection from “self.” All “X” indicate a lack of biological evidence for any synaptic connectivity in the specific pathway.

### 2.1. Model equations

The model in Figure 1 is defined by the set of first order differential equations in Equations (1)–(8). The neurotransmitter concentration in the synaptic cleft ([*T*]) is a function of the mean membrane potential of the pre-synaptic population, *V*_*pre*_, and is simulated with a sigmoid defined in Equation (1):

(1) $$\begin{array}{ll} \left[T\right]\left(V_{pre}\right)=\frac{T_{max}}{1+e^{-\frac{V_{pre}-V_{thr}}{\sigma }}},\; & \; \end{array}$$

where *V*_*thr*_ is the threshold voltage when the neurotransmitter concentration crosses the 50% of its maximum value *T*_*max*_, and σ is the steepness parameter of the sigmoid. An increase of neurotransmitter concentration in the synaptic cleft can be simulated in the model by decreasing *V*_*thr*_ and increasing σ.

Equation (2) defines the dynamics of the ionotropic synapses in the model viz. those mediated by the AMPA and GABA_*A*_ neuro-receptors. The variable *r* defines the proportion of open ion-channels on the post-synaptic population caused by the binding of the glutamatergic and GABA-ergic neurotransmitters with the AMPA and GABA_*A*_ neuro-receptors respectively.

(2) $$\begin{array}{ll} \frac{dr\left(t\right)}{dt}=\alpha \cdot \left[T\right]\left(V_{pre}\right)\cdot \left(1-r\left(t\right)\right)-\beta \cdot r\left(t\right),\; & \; \end{array}$$

where α and β refer to the forward and reverse rates of chemical reactions respectively.

Equations (3)–(5) define the dynamics of the metabotropic synapses in the model viz. those mediated by the GABA_*B*_ neuro-receptors. They activate G-proteins which in turn act as the “secondary messengers” and initiate the opening of ion channels.

(3) $$\begin{array}{r} \frac{dR\left(t\right)}{dt}=\alpha_{1}\cdot \left[T\right]\left(V_{pre}\right)\cdot \left(1-R\left(t\right)\right)-\beta_{1}\cdot R\left(t\right) \end{array}$$

(4) $$\begin{array}{r} \frac{d\left[X\right]\left(t\right)}{dt}=\alpha_{2}\cdot R\left(t\right)-\beta_{2}\cdot \left[X\right]\left(t\right) \end{array}$$

(5) $$\begin{array}{r} r\left(t\right)=\frac{{\left[X\right]}^{n}\left(t\right)}{{\left[X\right]}^{n}\left(t\right)+K_{d}} \end{array}$$

where *R* is the fraction of activated GABA_*B*_ receptors, which acts as a catalyst in activating the secondary-messenger G-protein (guanine nucleotide binding proteins); [*X*] is the concentration of the activated G-protein; *r* is the fraction of open ion channels caused by binding of [*X*] with independent binding sites; α_1, 2_ and β_1, 2_ are the forward and reverse binding rate constants respectively; *n* is the number of bound receptor sites and K_*d*_ is the dissociation constant of binding of [*X*] with the ion channels.

The resulting post-synaptic current, *I*_*psc*_(*t*), is defined in Equation (6):

(6) $$\begin{array}{ll} I_{psc}\left(t\right)=C_{uvw}\cdot g_{max}^{syn}\cdot r\left(t\right)\cdot \left(V_{psp}\left(t\right)-E_{rev}^{syn}\right),\; & \; \end{array}$$

where $g_{max}^{syn}$ and $E_{rev}^{syn}$ are the maximum conductance and reverse potential respectively and their values depend on the mediating synapse *syn*∈{AMPA, GABA_*A*_, GABA_*B*_}; *V*_*psp*_ is defined in Equation (7) and is the ensemble post-synaptic membrane potential; *C*_*uvw*_ is a normalized figure that represents the percentage of the synaptic contacts made on the post-synaptic cell population *u* by the pre-synaptic cell population *v*, and *w* represents the sign of the synapse i.e., excitatory or inhibitory.

(7) $$\begin{array}{ll} \kappa_{m}\cdot \frac{dV_{psp}\left(t\right)}{dt}=-\sum I_{psc}\left(t\right)-I_{leak}\left(t\right),\; & \; \end{array}$$

where κ_*m*_ is the ensemble membrane capacitance of the post-synaptic cell population,

The parameter I_*leak*_ in Equation (7) is the ensemble membrane leak current of the post-synaptic cell population and is defined in Equation (8):

(8) $$\begin{array}{ll} I_{leak}\left(t\right)=g_{leak}\left(V_{psp}\left(t\right)-E_{leak}\right),\; & \; \end{array}$$

where *g*_*leak*_ and *E*_*leak*_ are conductance and reverse potential respectively corresponding to “non-specific” leak (Golomb et al., 1996; Suffczyński et al., 2004) in the ensemble membrane of the post synaptic cell population.

### 2.2. Model parameterization

The model input is simulated by computer generated random noise that has a low variance and a white frequency spectrum. The resting state membrane potential of both excitatory cell populations of the model viz. the retinal cells and TCR are set to −65 mV. Thus, the mean of the retinal noisy input is −65 mV and the standard deviation is set to 2 mV^2^ to reduce the stiffness of the solution (in Matlab) for the set of differential equations defining the model in Equations (1)–(8).

The base values of the synaptic connectivity parameters in the model are based on physiological data (Van Horn et al., 2000; Sherman and Guillery, 2001; Jones, 2007) and mentioned in Table 2 along with the corresponding nomenclature for specific connectivities used in this work. For the purposes of this work in simulating the LGN dynamics of a de-corticated thalamus (discussed in Section 1), we ignore all afferent cortico-thalamic inputs to the model. A brief overview of the literature survey on intra-thalamic cell connectivity is provided below:

- *TCR afferents*: Data from the dorsal cat LGN (LGNd) (Van Horn et al., 2000) suggest that the TCR receive ≈ 7.1% of their inputs from the retinal ganglion cells (*C*_*tre*_), while ≈ 30.9% of their inputs are from inhibitory sources viz. IN and TRN. However, and to the best of our knowledge, there is no data available that distinguish between the afferent synaptic terminals from the IN and TRN. Thus, in this work, the GABA_*A*_ afferents from TRN and IN ($C_{tni}^{a}$ and *C*_*tii*_ respectively) and the GABA_*B*_ afferent from the TRN ($C_{tni}^{b}$) are tuned so that the sum total of all inhibitory afferents on the TCR is 30.9%. The remaining ≈ 62% of the connections are from the cortex as well as other sub-cortical sources, and are ignored in the present work for brevity.
- *IN afferents*: A study made on the cat LGNd in 1991 suggest that the IN cells receive around 25% synapses from the retinal spiking neurons, 37% from other inhibitory sources including themselves, while 26% synapses are from the cortex. However, according to a more recent study (Van Horn et al., 2000), these figures are reported as 47.4, 23.6, 29% respectively. On the other hand, data from the LGNd of a squirrel monkey (primate) indicates that the IN cells receive an equal proportion of each of the three categories of synaptic terminals (Jones, 2007). To maintain consistency with the source of data for TCR population, we follow the data by Van Horn et al. (2000) and set the retinal input (*C*_*ire*_) and self-inhibitory (*C*_*isi*_) connectivities in the IN as 47.4 and 23.6% respectively. It may be noted that the IN circuitry in the LGN has a unique triadic spiking arrangement (Sherman, 2004) consisting of dendrites that are not only post-synaptic to the retinal cells but are also pre-synaptic to the TCR cells as well as to themselves (dendro-dendritic synapses). These are referred to as the F2 terminals of the IN while the usual pre-synaptic axonal terminals are referred to as F1. However, the exact distribution of F1 and F2 terminals are not yet available from physiological studies; thus the above-mentioned figures for synaptic afferents to the IN refer to the combined numbers of both types of terminals.
  Experimental observations of IN cell dynamics do mention an excitatory feed from the TCR to these cells (Crunelli et al., 1988; Zhu et al., 1999a; Lörincz et al., 2008), however, these were speculations based on cell behavior as opposed to cell physiology. On the other hand, experimental studies on IN physiology suggest two specific cell types in the LGN (Cox et al., 2003): the intra-layer IN cells that do not receive any afferents from the TCR cells; the inter-layer IN cells that do receive inhibitory feedback from the TCR cells, and are often thought to be “stray” cells of the TRN. In the present work, we consider the intra-layer IN cell population only; thus, as in our previous work (Bhattacharya et al., 2011b), the IN population do not receive any synaptic afferents from the TCR.
- *TRN afferents*: Both thalamocortical and corticothalamic synapses on the TRN sector corresponding to the rat LGNd and visual cortex are excitatory (glutamatergic) in nature and constitute ≈ 30–40% and ≈ 50% respectively of the total synapses; the remaining up to 25% of the synapses are from other inhibitory sources including neighboring intra-population cells (Jones, 2007). In our model, we maintain the TCR afferent connectivity (*C*_*nte*_) as 35%, and self inhibitory connectivity (*C*_*nsi*_) as 20%.

The parameter [*T*]_*max*_ in Equation (1) is well approximated by 1 mM (Destexhe et al., 1998), while the base values for *V*_*thr*_ and σ are obtained by trial simulation studies on the model (further elucidated in Section 3.1) and are set to −32 and 3.8 mV respectively. The capacitance κ_*m*_ is set at 1 μF/cm^2^.

It is worth noting that while the model output time-series in Bhattacharya (2013) demonstrates rich dynamics corresponding to alterations in AMPA and GABA_*A*_ synaptic parameters and conforming to experimental observations, the period of oscillations in the model were below that of normal brain oscillations as seen in LFPs and EEGs. Appropriate modifications are made in this work to overcome the limitations of the previous work, and the parameters α, β and $g_{max}^{syn}$ in the Table 1A as well as *g*_*leak*_ in Table 1B reflect the modified unit scales.

The variable *V*_*psp*_ for TCR, TRN, and IN are initialized to the respective resting state values as in Table 1B; the variables “*r, R, X*” in Equations (2)–(5) are initialized to an arbitrarily small value 0.001 (and the ODE solutions do not show any dependency on the initial values). Please see the Table 1 legend for further information on parameter sources.

### 2.3. Simulation methods

The ODEs are solved using the 4^*th*^/5^*th*^ order Runge-Kutta-Fehlberg method (RKF45) in Matlab for a total duration of 40 s at a resolution of 1 ms. The output voltage time series is averaged over 20 simulations, where each simulation runs with a different seed for the noisy input. For frequency analysis, an epoch from 10 to 39 s of the output signal is sampled every 1 ms (1000 Hz) and bandpass filtered between 1 and 100 Hz with a Butterworth filter of order 10. The filtered signal is then transformed using 4-point FFT and power spectral density derived using the Welch periodogram. The power plots in Section 3 show the averaged power spectral density over 20 simulations. The bar plots show the total frequency content within each of the four frequency bands viz. delta (1–3.5 Hz), theta (3.75–7.5 Hz), alpha (7.75–13.5 Hz), and beta (13.75–20). Short Time Fourier Transform (STFT) is carried out on the averaged membrane potential of each cell population with a Hamming window of duration 1 s and overlap of 50%.

Simultaneous variation of parameters is an inherent feature in the brain as in all dynamical systems in nature. The kinetic modeling approach based NMM is computationally efficient in terms of both time and memory usage compared to the alpha-function based NMMs, as well as possessing greater biophysical plausibility. This approach is adopted in model presented here and has allowed for extensive simulation trials of the model involving simultaneous parameter variation in order to set the base parameter values in Table 1, as well as to study model behavior as discussed in the following text.

## 3. Results and discussion

The results presented herewith make a comparative study on the effects of incorporating the IN in the LGN model with the case when it is excluded from the circuit. In Sections 3.1–3.4, we present and discuss parametric deviations that are observed to effect a state transition in the model output time-series and a shift in its power spectra. In Section 3.5, we examine a case study by simulating SSVEP in the model.

### 3.1. Causality of the neurotransmitter concentration: setting base parameters

**Figure 2:**
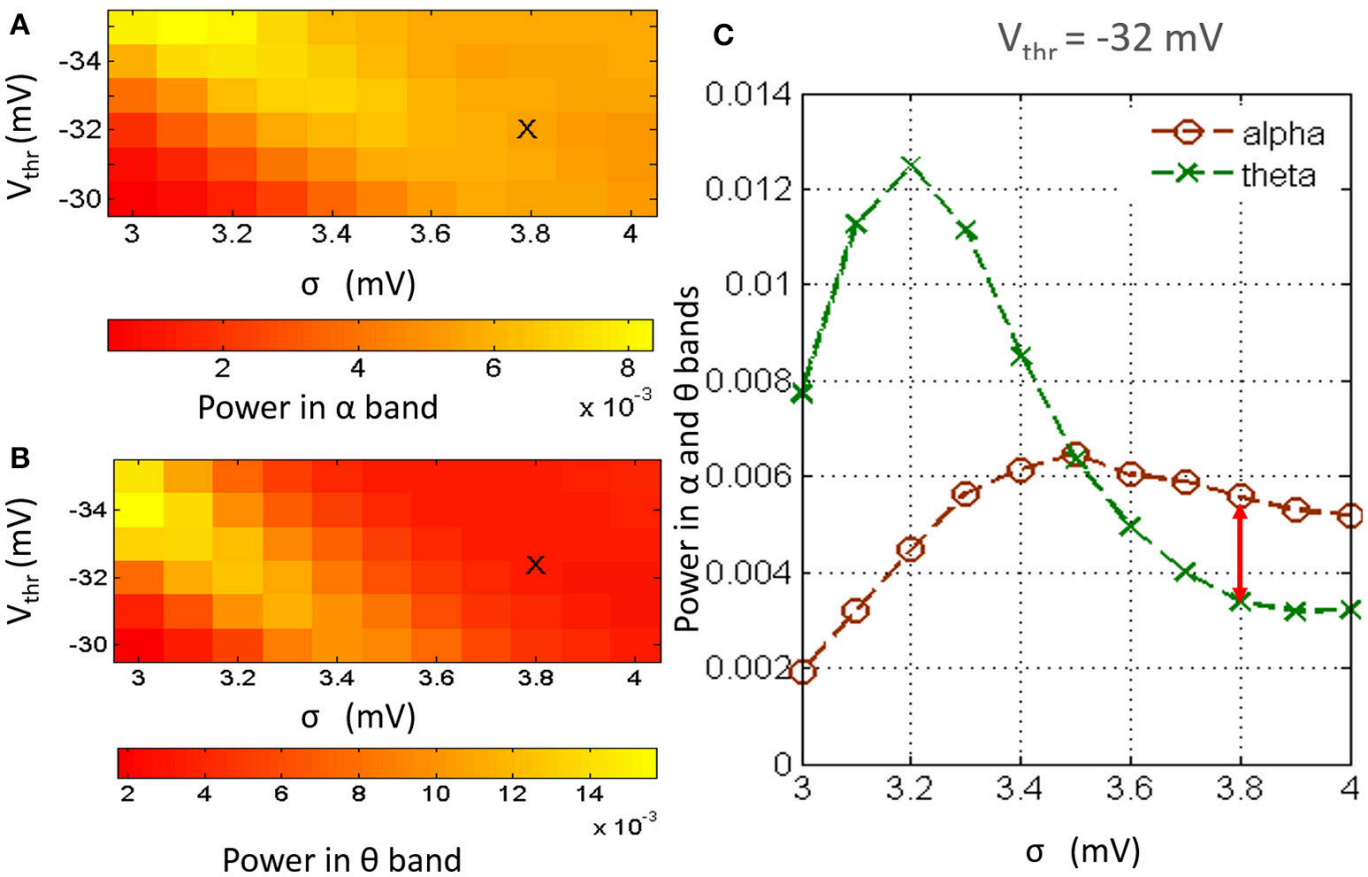
**The model output power spectra for simultaneous variation of parameters ***V***_***thr***_ and σ and within the (A)** alpha and **(B)** theta frequency bands. **(C)** Histogram of the model output power within the theta and alpha bands at *V*_*thr*_ = −32 mV and for values of σ between 3 and 4 mV at a resolution of 0.2 mV. The power within the alpha band is greater than that within the theta band for σ ⩾ 3.6. For 3.6 ⩽ σ ⩽ 4, the alpha peak has a maximal difference with the theta peak at σ = 3.8 mV. Thus, the base values of *V*_*thr*_ and σ are set at −32 and 3.8 mV respectively.

Figures 2A,B show an overview of the model output power within the alpha and theta bands respectively for simultaneous variation of σ in the range 3–4 mV at a resolution of 0.1 mV, and *V*_*thr*_ in the range −30 to −35 mV at a resolution of 1 mV, simulating a gradual increase of neurotransmitter concentration [*T*] (the reader may please refer to Equation 1). The power within the theta band has a left skew for lower values of both parameters; there is a drop in the overall band power for increasing values of *V*_*thr*_. Power within the alpha band saturate for higher values of σ across all values of *V*_*thr*_; however, the absolute power is less than that within the theta band for lower values of *sigma*. In general, for a fixed value of *V*_*thr*_ (σ), increasing (decreasing) σ (*V*_*thr*_) causes an increase in [*T*] and effects a progressive shift of the model output power spectra from a dominant theta band power to a dominant alpha band power accompanied by a consistent increase in beta band power.

As mentioned in Section 2.2, the base values of both parameters *V*_*thr*_ and σ are set by visual inspection of the results in Figure 2 such that the model output power is dominant within the alpha band. In Figure 2A, the image show minimal skewness for *V*_*thr*_ = −32 mV. In Figure 2C, the case for *V*_*thr*_ = −32 mV is shown: we note that at σ = 3.8, the alpha band power is greater than that of the theta band, and the difference between the two band powers is maximum for the tested range; thus, *V*_*thr*_ = −32 mV and σ = 3.8 mV are set as base parameter values in the model.

Discussion

The gradual alpha to theta band transition with reduced levels of neurotransmitter concentration in the model mimics the EEG marker of transition from a state of wakefulness to a state of drowsiness (Hughes and Crunelli, 2005). It is now well understood that the release of neurotransmitters in the synaptic cleft is mediated by calcium current; an in-depth study by Llinas et al. (1981) on squid giant axon suggests transmitter depletion in the cleft due to reduced pre-synaptic activity, which in turn lead to reduced post-synaptic activity. In the LGN, McCormick (1992) have demonstrated that thalamic slices *in vitro* display membrane depolarization and cessation of rhythmic oscillations on application of cholinergic and noradrenergic neuromodulatory inputs. This implies that conversely, depletion of these neuromodulators may lead to slow synchronous rhythms in the LGN. More recently, Zhao et al. (2002) have shown that dopaminergic neuro-receptors affect information transmission efficiency in the LGN by neuromodulation of the IN cells. Furthermore, their study suggest a stronger suppression of the TCR cells with blockage of GABA_*A*_ neuro-receptors. Our model-based study show an abrupt (as opposed to progressive) transition from alpha to theta rhythm when the IN feed-forward inhibition of the TCR cells are deleted in the circuit (see Section 3.2). Overall, it may be speculated that the state transition in the model may mimic the effect of IN cells due to neuromodulatory influences from extra-thalamic sources.

### 3.2. Causality of the interneurons in the LGN circuit

**Figure 3:**
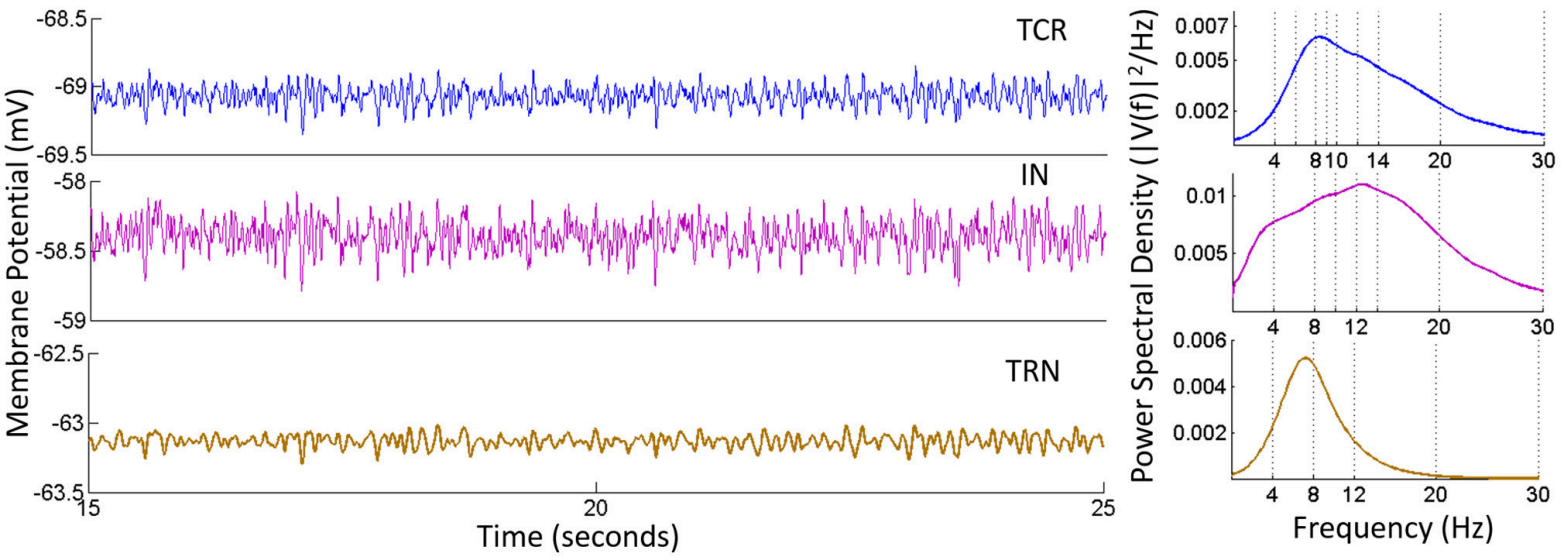
**Time-series (Left) and Power spectral density (Right) plots for the TCR, IN and TRN cell populations corresponding to base parameter values in the model**. The dominant frequency of oscillation of both TCR and IN is within the alpha band (8–13 Hz) with peaks at around 8.5 and 13 Hz respectively. Furthermore, both these populations show a wide power spectra spanning the theta (4–7 Hz), alpha and lower-beta (14–20 Hz) bands. The TRN shows a comparatively narrow power spectra spanning the theta and lower-alpha (5–10 Hz) band with a dominant peak within the theta band at ≈ 7.5 Hz. The time-series show-case a representative sample from 15 to 25 s of the total simulation time of 40 s.

**Figure 4:**
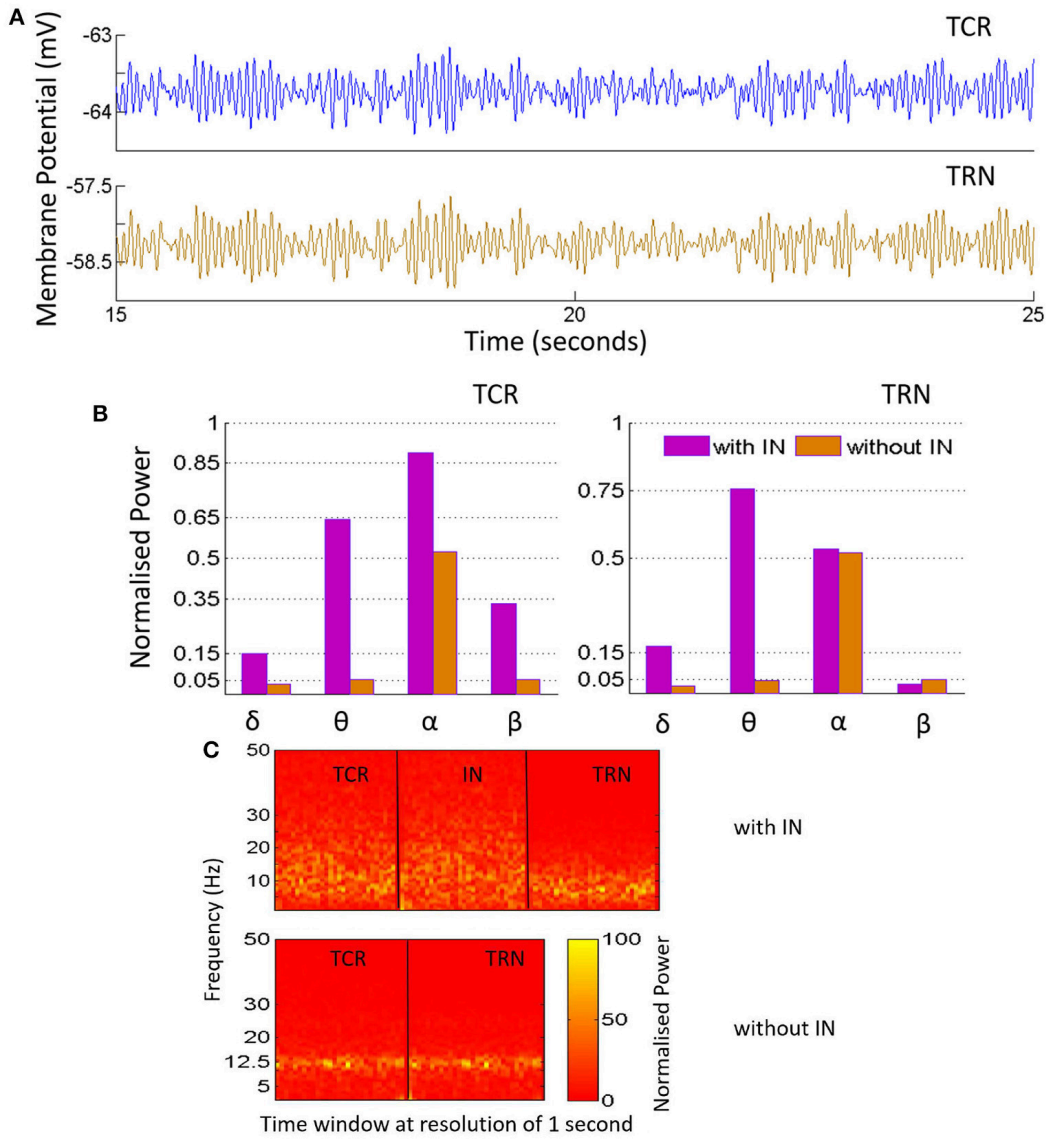
**(A)** The time-series plots show the phase-locked waxing-and-waning oscillatory patterns within the alpha band, (hallmarks of EEG and LFP in a quiet awake state with eyes closed, and resting) in both TCR and TRN, and in the absence of IN in the circuit. The average membrane potential from 15 to 25 s of the total simulation time of 40 s is show-cased and may be compared to the time-series plots in Figure 3, corresponding to when the IN is included in the circuit. **(B)** The bar plots for TCR (Left) and TRN (Right) show a comparison between when the IN is in the circuit and when the IN is disconnected: In the former case, the TCR and TRN have different power levels within the specified frequency bands. The dominant frequency of the TCR is within the alpha band, while the dominant frequency in the TRN is within the theta band. In the latter case, i.e., with the IN is removed from the circuit, the proportion of power within the delta, theta and beta bands in both TCR and TRN are negligible in comparison to the power within the alpha band. **(C)** The STFT plots in the lower panel (without IN) show a fairly stationary signal for both TCR and TRN with a peak at ≈ 12.5 Hz in the absence of IN. In comparison, when the IN is included in the circuit, the STFT plots (upper panel) for the TCR indicate a broad power spectra spanning the theta, alpha and lower-beta bands and is similar to that of the IN. However, STFT plots for the TRN output lies within the theta to lower-alpha region (4–10 Hz), implying suppressed dynamics in the presence of IN.

The time series and power spectra of the model cell populations when all parameters are at their base values are shown in Figure 3. The membrane potential of the TRN has a low peak-to-peak oscillatory envelope of ≈ 0.3 mV in comparison to ≈ 0.8 mV for TCR and IN. The power spectra of both TCR and IN are broad with peak frequencies at ≈ 8.5 and 13 Hz respectively. The corresponding STFT plots in Figure 4 (**C**: with IN) show that the power spectra of both populations span the theta, alpha and lower-beta (4–20 Hz) frequency bands. In comparison, the TRN has a sharp power spectral peak at around 7.5 Hz, and the STFT plot in Figure 4 (**C**: with IN) show a narrow power spectra within the theta to lower-alpha (4–10 Hz) region corresponding to base model parameter values.

Next, the IN cell population is disconnected from the network by making *C*_*tii*_ = 0. This condition simulates the blocking of GABA-ergic feed-forward inhibition of the TCR due to neuromodulatory effects on the IN by several neurotransmitters such as acetylcholine (McCormick, 1992) and dopamine (Zhao et al., 2002). The time series output of both TCR and TRN in Figure 4A shows a distinct bifurcation with synchronized waxing-and-waning of amplitude within the alpha band frequency. The mean membrane potential of both populations show depolarization and increased peak-to-peak oscillation compared to their respective counterparts in Figure 3. The bar plots of both TCR and TRN in Figure 4B demonstrate the dramatic decrease in theta band power and the distinct dominance of alpha band power when the IN is removed from the circuit. The STFT plots for both TCR and TRN in Figure 4 (**C**: with IN) indicate a stationary power spectra with a dominant frequency within the alpha band. Furthermore, in the absence of IN, decrease of neurotransmitter concentration in the model effects a dramatic slowing of the model output: thus, while the time series still retain the amplitude-waxing-and-waning pattern in synchrony with the TRN, the dominant frequency of oscillation is within the theta band (not shown here; please refer to discussion in Section 3.1).

Discussion

The model results show a distinct suppression of TRN activity in the presence of IN in the circuit; the corresponding time series is low-amplitude with a broad power spectral content spanning the alpha to lower-beta regions, similar to EEG and LFP in awake cognitive state. Removal of IN effects feed-forward disinhibition of the TCR and generation of synchronous waxing-and-waning oscillations. Experimental studies have shown that the waxing-and-waning patterns in EEG, both in alpha rhythms (amplitude) as well as in sleep spindles (frequency), are the result of the feed-forward and -back interactions between the TCR and the TRN. As both patterns underpin a state of “low vigilance” (Saalmann and Kastner, 2011) i.e., devoid of task-related activity and reduced information transmission efficacy in the retino-geniculo-cortical pathway, it is suggested (Saalmann and Kastner, 2011) that a state of increased information transmission (as opposed to a state of low vigilance) is facilitated in the circuit by suppressing the inhibitory influence of the TRN. The model validates these experimental observations and further predicts that feed-forward inhibition from the IN play an important role in cognitive activities by suppressing the feedback inhibitory effects of the TRN cells.

Another interesting observation by Halassa et al. (2014) reports better task-related performance when the inhibitory efferents of the TRN are suppressed by application of an “external stimulus”—we hypothesize that the “external stimulus” may have simulated the indirect effect of IN on the TRN. Overall, the model predictions underpin a causal role of the IN in transitions between awake cognitive state to that of diminished cognition.

In addition, the model results show that common mechanisms in the LGN cause alpha to theta band transitions, thus agreeing with experimental observations suggesting that common neuronal mechanisms underlie EEG and LFP of alpha and theta rhythms (Hughes et al., 2004).

### 3.3. Varying the connectivity parameters

**Figure 5:**
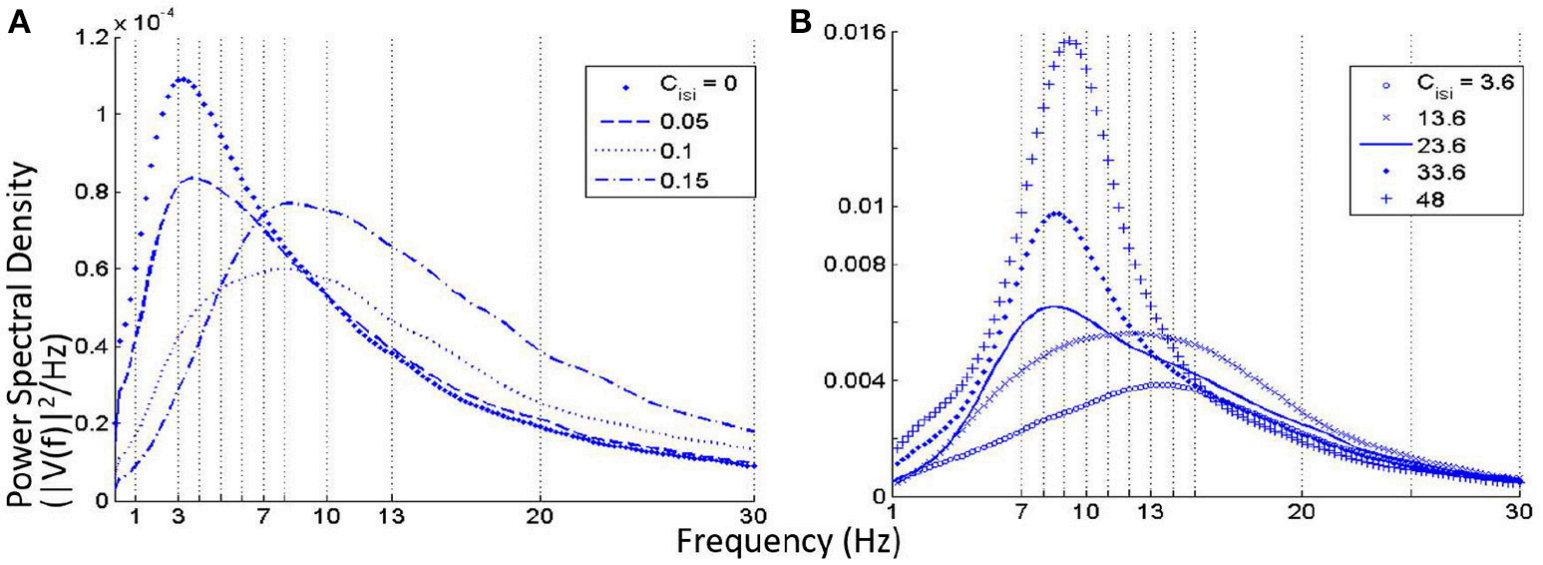
**(A)** Near zero values of the self-inhibitory connectivity of the IN population (*C*_*isi*_) show dominant frequency within the theta and delta bands. **(B)** For increased values of *C*_*isi*_ from its base value (23.6), the power spectra is fairly flat with dominant frequency within the alpha and beta bands. For decreasing values of the parameter, there is an increase of power within the alpha band with a slight right-shift of the peak frequency.

**Figure 6:**
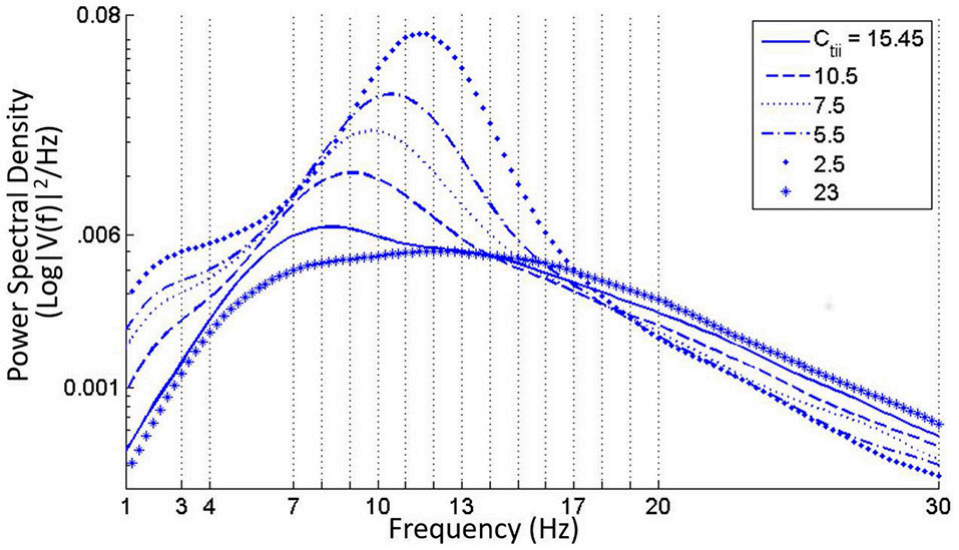
**Increase in power within the alpha band with diminishing values of the feed-forward inhibitory connectivity of the IN on TCR population (***C***_***tii***_)**. For values less than around 5, the time-series shows synchronized oscillation of the TCR and TRN. For values higher than the base parameter value of 20, the power spectra is fairly flat with maximum power within the alpha and lower beta bands.

**Figure 7:**
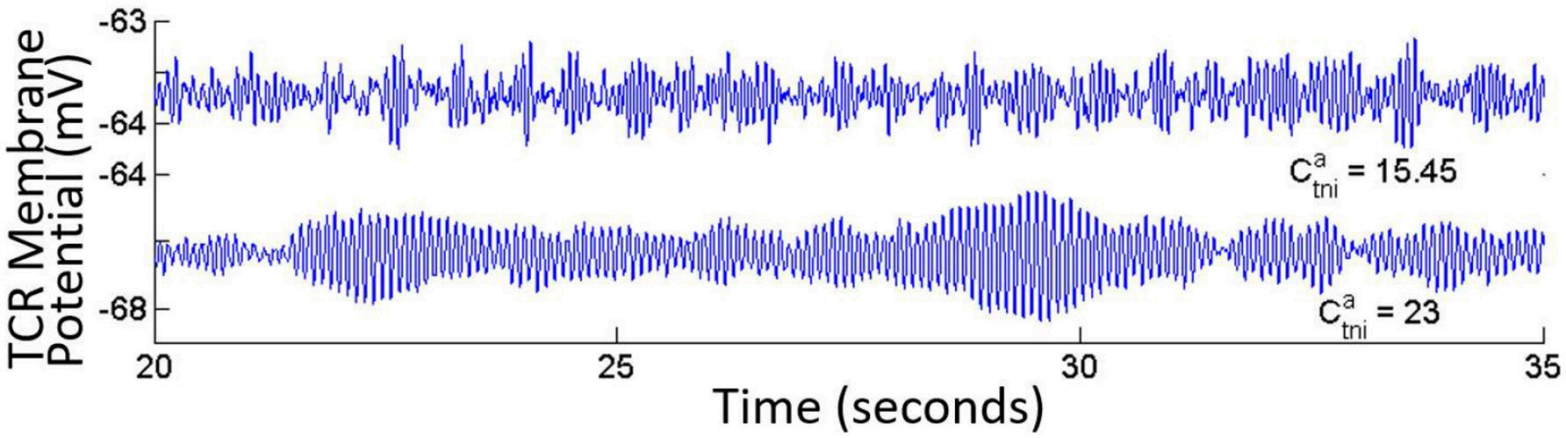
**The TCR population output displays alpha band waxing-and-waning behavior in synchrony with the TRN population in the absence of IN in the circuit**. Under this condition, if the ionotropic GABA_*A*_ connectivity from the TRN to the TCR ($C_{tni}^{a}$) is increased, the model time-series shows a bifurcation into a limit cycle mode. With the IN population incorporated in the LGN circuit, variations of $C_{tni}^{a}$ values do not have any significant effect on the output.

**Figure 8:**
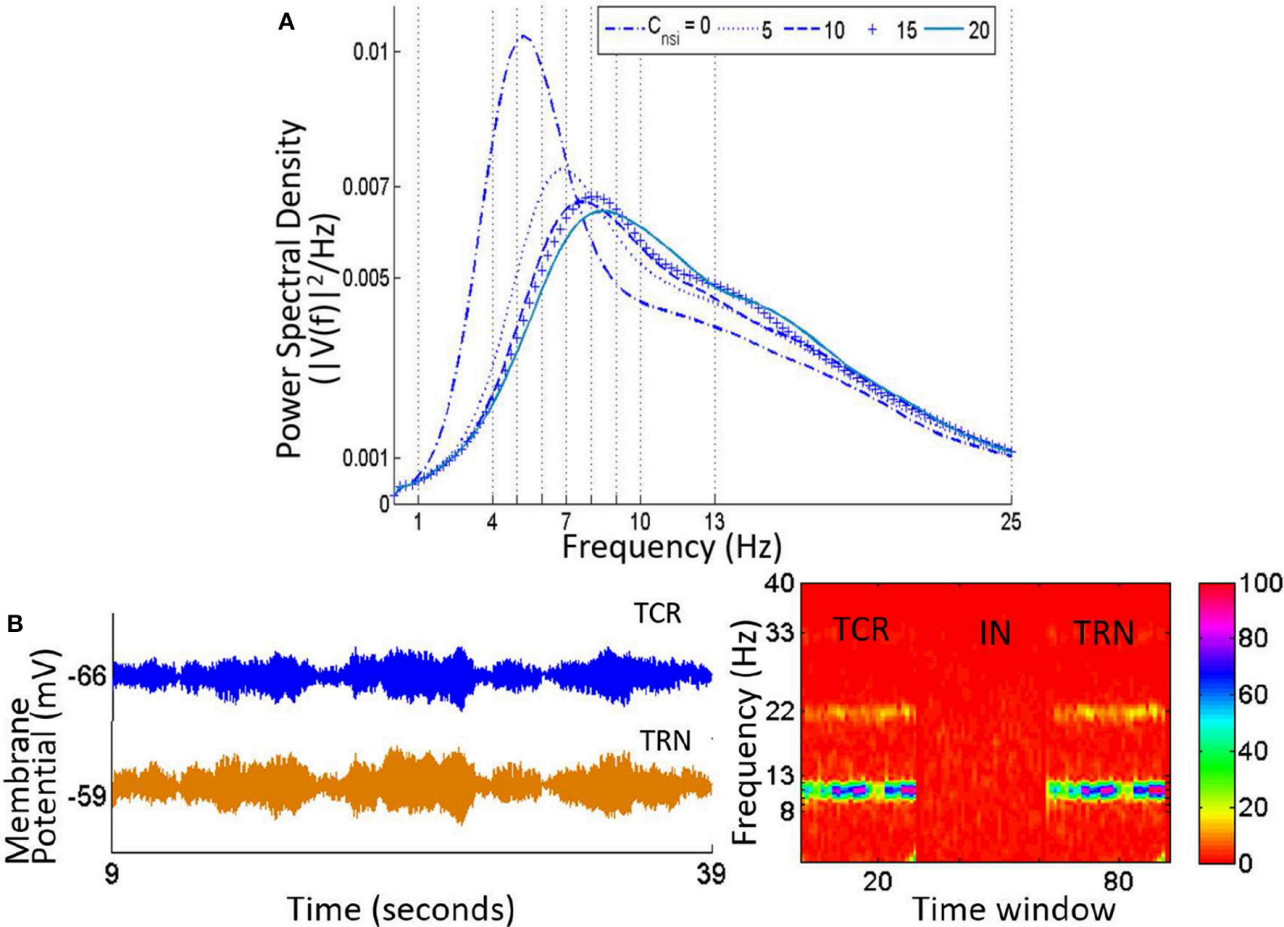
**(A)** Slowing of the power spectra with reduced value of the self-inhibitory pathway (*C*_*nsi*_) of the TRN population. **(B)** In the absence of IN in the LGN circuit, the TCR and TRN population output are synchronized and oscillate in the limit cycle mode for reduced values of *C*_*nsi*_. The figure shows the case when *C*_*nsi*_ = 10.

The synaptic connectivity parameters in the model are varied around their base values, the details of which are mentioned in Table 3 along with a brief overview about their effects on the model behavior. The results are presented and discussed below:

- When the self inhibition connectivity parameter of the IN (**C**_**isi**_) is decreased, the TRN population output is suppressed significantly in spite having no direct connectivity with the IN cells. Overall power in the model output is decreased due to increased inhibition from the IN population, and the power spectra shifts right with relatively more power within the upper alpha (10–13 Hz) and lower beta bands (14–20 Hz) as shown in Figure 5B. Increasing the parameter values above the base value reduces the effect of IN on the TCR, and thereby on the TRN. A narrow peak within the alpha band in Figure 5B indicates an increased effect of the TRN population on the TCR cells.
  For *C*_*isi*_ ≤ 0.15 and as shown in Figure 5A, the overall power decreases with maximum frequency within the theta band, until *C*_*isi*_≈ 0.05%, when the dominant frequency shifts to the delta band. When *C*_*isi*_ = 0, the dominant frequency is within the delta band.
  Discussion
  Cortico-cortical and cortico-thalamic modulation is well known to be vital for delta band oscillations corresponding to slow wave sleep and/or reduced cognitive state. In the model, the only instance of delta band oscillations are simulated for the case corresponding to synaptic deletion of the self-inhibitory afferent pathway of the IN that effectively disinhibits the IN, thus increasing the feed-forward inhibition of the TCR.
  Based on these observations, we hypothesize that the cortical feedback to the IN cells target the self inhibitory mechanisms in the population, which in turn effects slow wave oscillations in the LGN. While it may not directly affect an alpha band oscillation, the cortical feedback may cause an increased self inhibition in IN cells, which in turn will lead to decreased effect on the TCR, thus allowing the TRN inhibition to dominate the output showing synchronous alpha rhythms.
- A progressive decrease of **C**_**tii**_ from its base value shows a progressive increase of power within both alpha and theta bands (Figure 6). For *C*_*tii*_ ⩽ 5, the TCR and TRN time-series display alpha rhythmic synchronous oscillations with amplitude waxing-and-waning. Increasing the parameter value did not have any significant effect and the model output showed a relatively flat spectrum within the alpha and lower beta bands, indicating an increased inhibition in the circuit; this is demonstrated in Figure 6 for *C*_*tii*_ = 23.
- Decreasing the retinal input connectivity for the IN (***C***_***ire***_) effects a significant rise of the alpha band power in the TCR output until below a threshold, when the TRN dominates and the output show alpha band waxing-and-waning oscillations. Increasing *C*_*ire*_ from its base value does not affect the model output.
  Discussion
  In summary, the results show that a decrease in feed-forward inhibition in the LGN causes an increase in power within the alpha and theta bands, implying that at base parameter values, the inhibitory effect of TRN on TCR is suppressed by IN. We have discussed (above) the correspondence between cognitive states and suppression of TRN activity by the IN. Indeed, experimental evidence suggest that the IN feed-forward inhibition serves to enhance the “sensitivity to visual features” (Wang et al., 2011a), and thus increase the overall efficiency of retino-geniculo-cortical information transmission (Dublin and Cleland, 1977; Hirsch et al., 2015). The results support these experimental reports.
  The model identifies a vital difference between the two GABA-ergic efferent pathways of the IN—synaptic variation in the efferent pathway to the TCR (*C*_*tii*_) bears a linear relation to the power spectra, unlike in the self inhibitory efferent pathway (*C*_*isi*_), where synaptic depletion/enhancement shows a non-linear relation with the power spectra dominant frequency. Thus, it seems likely that these two pathways form parts of different circuitry in the LGN: while the *C*_*isi*_ may be a part of the cortico-thalamic feedback mechanism (discussed above), the *C*_*tii*_ appears to be an intra-thalamic connectivity that aids retino-geniculo-cortical information transmission and regulates brain cognitive states. This hypothesis is further supported by identical effects of synaptic depletion in both the retino-geniculate (*C*_*ire*_) and the intra-geniculate (*C*_*tii*_) pathways, causing amplitude waxing-and-waning synchronous oscillations within the alpha band in both TCR and TRN, implying a reduced cognitive state.
- The GABA_*A*_ inhibitory feedback from the TRN to the TCR (${\text{C}}_{\text{tni}}^{\text{a}}$) shows minimal effect on model output. However, there is a dramatic effect when the IN is disconnected from the network: The time-series of both TCR and TRN display bifurcation into a high amplitude alpha band limit cycle oscillations for $C_{tni}^{a}>$ its base value (Figure 7). The limit cycles disappear for $C_{tni}^{a}⩽15.45$ (base value), and the output signal displays synchronous waxing-and-waning alpha band oscillations (as is expected from our earlier observations with disconnecting the IN from the network).
- Decreasing the self inhibitory connectivity of TRN (***C***_***nsi***_) from its base value of 20 cause a small but progressive increase of power within the theta band as shown in Figure 8A. However, removing IN from the network with a simultaneous decrease in parameter value induces a bifurcation of the time-series to a limit cycle mode for both TCR and TRN; the case for *C*_*nsi*_ = 10 is shown in Figure 8B, left panel. The corresponding STFT plots in Figure 8B, right panel show second harmonic of the dominant frequency within the alpha band. For both cases i.e., with and without IN, when *C*_*nsi*_ = 0, the dominant frequency of oscillation is within the theta band as seen in Figure 8A.
  Discussion
  The results are in agreement with the suppression of the TRN output in presence of the IN as discussed in Section 3.2: efferent and afferent synaptic pathways in the TRN in the model have minimal effect when the IN has the dominant inhibitory influence in the LGN circuit. Bifurcation to synchronous waxing-and-waning of amplitude as well as limit cycle oscillations are observed in both TCR and TRN when the IN is absent from the LGN circuitry. Limit cycle oscillations are often associated with abnormal brain behavior for example epilepsy (Suffczyński, 2000). Thus, the model predicts a homeostatic role of the IN in the LGN circuitry by controlling high amplitude synchronous oscillations.
  In Saalmann and Kastner (2011), the authors speculate that a disinhibition mechanism adopted by the TRN (by increasing its self inhibitory connectivity) may regulate the appearance/absence of synchronous oscillations in the TCR. However, the model predicts the IN as the “master switch” that “enable” such mechanisms. When the IN synaptic pathway is sufficiently depleted, the IN is “disabled” in the circuitry, and both inhibitory efferent pathways of the TRN show significant role in TCR oscillations. In addition, the model predicts that with deletion of the self inhibitory mechanism in the TRN cells, the TCR output is “stalled” within theta band, an EEG biomarker of drowsy states or reduced cognition. This is contrary to the speculations made in Saalmann and Kastner (2011), where the authors suggest an increased self inhibition in the TRN population as a possible mechanism for facilitating useful thalamocortical information transmission. The disagreement may be due to the lack of a cortico-thalamic pathway in the model, and will need further investigation in future works with enhanced thalamo-cortico-thalamic circuitry.

**Table 3:** **Synaptic connectivity parameters that show sensitivity to the IN in the model are increased (>) and decreased (<) progressively with reference to their respective base values**.

**Parameter**	← <		**Base value**	> →	
*C*_*isi*_	3.6, 13.6		23.6	33.6, 40	α ↑
	0.1, 0.15	θ ↑			
	0, 0.05	δ ↑			
*C*_*tii*_	2.5, 5.5, 7.5, 10.5	α ↑	15.45	23, 30.9	
	< 5	α_*aww*_			
*C*_*ire*_	7.4, 27.4, 37.4	α ↑	47.4	57.4, 67.4	
$C_{tni}^{a}$	0, 2.5, 7.5, 11.6	α_*aww*_ (without IN)	15.45	23, 30.9	limit cycles (without IN)
*C*_*nsi*_	0, 5, 10, 15	θ ↑	20	25, 30	

The parameter values mentioned in the table are representative samples of the range of values for which the model is tested in this work. An overview of the significant effects on the model output time-series and power spectra corresponding to the respective parameter variation are also mentioned where ↑ indicates an increase, and ↓ indicates a decrease of power within the specified frequency band. All synchronous waxing-and-waning oscillatory patterns within the alpha band are indicated as α_aww_, where the suffix “aww” refers to the amplitude waxing-and-waning pattern.

### 3.4. Effects of leak conductance

Increased or suppressed potassium leak currents are reported as facilitating hyperpolarization and depolarization respectively of cell membrane potential, and show evidence of the critical role in normal (Goldstein et al., 2001) and abnormal brain-state transitions (Gentiletti et al., in press). Neurotransmitters/neuromodulators such as serotonin and acetylcholine inhibit potassium leak currents to enhance neural excitability. On the other hand, anaesthetics are known to decrease excitability in muscles by increasing leakage conductance. To make a qualitative validation of these observations in our model, we vary the leakage conductances of all three cell populations in the model.

The *g*_*leak*_ for any one cell population is increased to 100 μS/cm^2^ progressively, while the values of the same for the other two cell populations remain at their base values of 10 μS/cm^2^. A simultaneous variation of neurotransmitter concentration parameter [*T*] is also made by varying σ. The results are summarized below:

- When *g*_*leak*_ = 100 μS/cm^2^ for the **IN** cells, their mean membrane potential is hyperpolarized, causing a reduced effect on TCR and TRN, both of which show a depolarization. However, for reduced values of the neurotransmitter concentration (σ < 3.4), the TCR cells are hyperpolarized.
- When *g*_*leak*_ = 100 μS/cm^2^ for **TRN** cells, their mean membrane potential is hyperpolarized and the TCR cells are depolarized, and a smooth alpha to theta transition occurs with decreasing neuro-transmitter concentration.
  However, if the IN is removed from the circuit, increasing *g*_*leak*_ for the TRN population causes a depolarization in both TRN and TCR cells. The time series of both cell populations (not shown here) display synchronous alpha rhythmic waxing-and-waning oscillations while the power spectra display harmonics of the dominant frequencies within the alpha band.
- When *g*_*leak*_ = 100 μS/cm^2^ for the **TCR** cells, both TCR and TRN are depolarized, with a larger beta content in the power spectra for increasing neurotransmitter concentration. Both time series and power spectra display smooth transition to lower frequency bands with decreasing neurotransmitter concentration. Removing the IN did not show any drastic change in the output characteristics.

Discussion

The results imply that for a model tuned to oscillate within the alpha band and consisting of all three LGN cell populations, hyperpolarization of the TCR is effected only when the leakage conductance of the IN population is increased, and under the condition of reduced transmitter concentration; we note that this is similar to the above-mentioned effects of anaesthetics (Goldstein et al., 2001). Furthermore, in their experimental investigation on interneuron physiological characteristics, Pape and McCormick (1995) mention that membrane hyperpolarization is affected by an increase in membrane potassium conductance, which in turn is effected by activating acetylcholine (ACh) receptors. All cell populations of the LGN are known to receive significant cholinergic inputs from the brainstem (Sherman, 2006), which play a dominant role in effecting alpha band oscillations in the LGN (Saalmann and Kastner, 2011). Thus, the effects of increased leak conductance in the model may be a simulation of the causality of brainstem cholinergic inputs to the LGN.

On the other hand, if the synaptic efferent from the IN to TCR is decreased while leak conductance of TRN is increased, both TCR and TRN are depolarized leading to abrupt state transitions to high amplitude synchronous oscillations with dominant alpha band frequency; decreased neurotransmitter concentration under these conditions generate synchronous waxing-and-waning within the theta band. This observation, once again, predicts a significant role of the IN in maintaining a homeostasis in the LGN; it minimizes any unwanted state transitions in the circuit that may be caused by increase of the leakage current.

Overall, our model-based study emphasize the role of leakage potassium currents in the LGN circuitry and identifies the need for further investigation and validation with experimental studies in the context of unwanted and abrupt transitions in the circuit.

### 3.5. Simulating steady state visually evoked potentials: a case study

As a case study, the LGN model tuned to simulate alpha rhythmic output in an awake state is tested for simulating SSVEP output corresponding to a periodic impulse train simulating visual stimulus. The white noise input to the model as used in Sections 3.1–3.4 is now superimposed by periodic impulse train of frequency *f* ∈ {5 − 50Hz}. The amplitude of the periodic impulses are set arbitrarily at a value of 10 mV.

**Figure 9:**
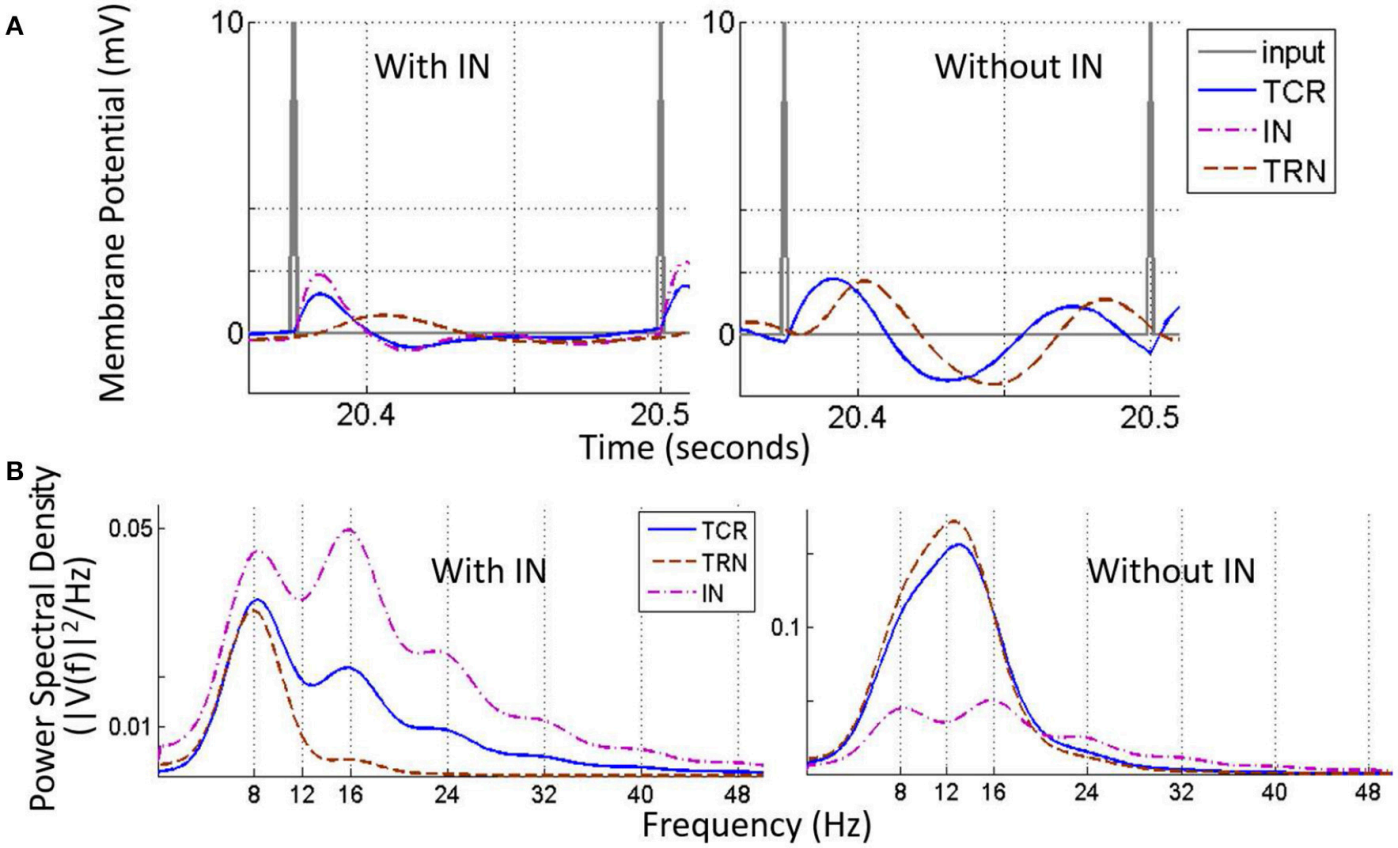
**The Steady state visually evoked potential (SSVEP) simulated in the model corresponding to an input periodic impulse train at 8 Hz superimposed on a white random noise**. **(A)** A representative sample of 1 cycle of the total simulation time is shown for all three cell populations. For clarity, the input signal is represented by the impulse trains only i.e., the white noise component is not shown. When the IN is in the circuit (left panel), the TCR and IN population have similar waveform with peaks that are in-phase; both waveforms settle to a steady state prior to the next cycle. The TRN population peak lags that of the TCR and IN by 20 ms. When the IN is disconnected from the circuit (right panel), the TCR waveform is now similar to that of the TRN population, and peaks at a delay of 10 ms compared to when receiving inhibitory afferents from the IN (left panel). **(B)** The power spectral density plot (left panel) for both TCR and IN show peaks at the fundamental frequency corresponding to that of the periodic impulse train, with harmonics at integral multiples of the fundamental frequency. However, for TRN, the peak frequency is at ≈ 7.5 Hz. With IN feed-forward synaptic pathway deleted from the circuit (right panel), both TCR and TRN population dominant frequency is at ≈ 13 Hz. This do not resemble the input impulse train frequency, rather reflect the inherent circuit frequency with white noise input (see Figure 4). The magnitude of power in the dominant frequency for both TCR and TRN are much higher compared to when IN was in the circuit (the power spectra for IN is included just for ease of comparison). Power within harmonics for both TCR and TRN are negligible relative to that within the dominant frequency.

Figure 9 shows the time series and power spectral density plots corresponding to an 8 Hz impulse train input:

With all parameters at their respective base values, the TCR and IN time-series are in-phase (Figure 9A, left) with a power spectral density (Figure 9B, left) peaking at the fundamental frequency 8 Hz (periodic input stimulus frequency) for TCR and at the second harmonic 16 Hz for IN. Furthermore, both power spectra show harmonics at integer multiples of the fundamental frequency. In comparison, the TRN time series show a low amplitude of oscillation and peak at a delay of around 20 ms from that of IN and TCR. The power spectral peak for TRN is at 7.5 Hz. (We note that across all frequencies (*f*) in the tested range for this study, the power spectral peaks for the TRN population consistently lie in the range of ≈ 6–8 Hz when the IN is included in the LGN circuitry).

Next, the IN is removed from the circuit; the amplitude of oscillation for the TRN time-series increases significantly in (Figure 9A, right), which is reflected in the increased magnitude within the dominant frequency in the power spectra (Figure 9B, right). The TCR time-series pattern changes dramatically and is now similar to that of the TRN, albeit at a lead of around 10 ms. The power spectral plot also follows that of the TRN with a peak frequency at 13 Hz, and thus do not reflect the that of the input impulse train. In general, across all *f* within the tested range and when the IN is removed from the circuit, both TCR and TRN show dominant frequency within the range 12–14 Hz, thus over-riding the fundamental frequency of the input impulse train. For some *f* across the tested range, the power spectra of TCR population show harmonics. However, the power magnitudes in these harmonics are negligible in most cases; where the TRN population power spectra show harmonics, these are always at integral multiples of the peak frequency in the range 12–14 Hz.

Discussion

A study by Babadi et al. (2010) suggests that around 33% of TCR cells in the LGN receive “locked” feed-forward inhibitory inputs from the IN; “locked” (“non-locked”) refers to the case when TCR and IN receive inputs from same (different) retinal ganglion cells (RGC). Furthermore, output of locked TCR cells bear a high degree of correlation with RGC inputs and is speculated to be a mechanism for increasing LGN response precision (Blitz and Regehr, 2005). In the model, both TCR and IN receive the same retinal input for a particular simulation, and the output of both populations are perfectly in-phase; thus the model output may be thought to simulate locked TCR response observed in experimental studies. While we did not consider this aspect (i.e., locked/non-locked IN cells) in our model design, the validation with experimental studies imply a degree of robustness in the model; however, this needs to be tested thoroughly prior to making claims.

The appearance of alpha rhythmic dominant frequency in the model output power spectra underpin the dominant role of TRN with abolition of IN inhibitory input to the TCR and is consistent with our previous results in Sections 3.2 and 3.3. Moreover, the disappearance of the input impulse frequency in the TCR power spectra indicates a de-correlation with the input; rather, the TCR power spectra is now correlated with that of the TRN. This is an interesting phenomenon in the model considering that the TCR continues to receive direct retinal input. These observations possibly imply a greater significance of the indirect sensory pathway of the TCR via its afferent pathway from the IN. Along these lines, synchronous oscillations in the LGN are suggested as a mechanism for decorrelating retinal input from the thalamic output in order to reduce the efficiency of information transmission in the geniculo-cortical pathway (Saalmann and Kastner, 2011). The model validates these speculations and further predicts that: the dominant role of the TRN is facilitated by what appears to be a separate circuit mechanism that inhibits the IN cells beyond a threshold, so that these are in a “disabled” mode and have minimal inhibitory influence on the TCR cells.

Another interesting implication of the results are the known symptoms of fatigue during SSVEP-based BCI experiments (Cao et al., 2014). The increased level of fatigue in a subject undertaking the experiment can drastically reduce the performance, and the corresponding EEG shows an increased proportion of alpha and theta band power. In our study, deleting the IN pathway to the TCR cells effect a significant rise in the power within both alpha and theta bands. This raises speculation that neuromodulatory circuits inhibiting the IN may play an indirectly role in inducing fatigue corresponding to SSVEP-based BCI tasks.

Overall, the preliminary results on the model simulation of SSVEP validates experimental research reporting significant role of IN in modulating visual input that is relayed to the cortex by the TCR population (Lörincz et al., 2009; Wang et al., 2011b; Hirsch et al., 2015). We hypothesize that any anomaly in the IN circuitry will disturb the normal processing of visual information during an awake cognitive state.

## 4. Conclusion

We use a neural mass computational model of the thalamic LGN, implemented with synaptic kinetics, to understand the effects of the inhibitory interneuron (IN) population on the LGN dynamics. The IN population constitute around 20–25% of all sensory information carrying thalamic nuclei in mammals, and in the LGN of rats. Specifically in the LGN, the IN cells receive around 47% of their inputs from the retinal spiking neurons. It is thus not surprising that extensive research have investigated the functional significance of the IN afferent and efferent synaptic pathways in precise spatial and temporal transmission of visual information to the cortex (see Hirsch et al., 2015 for a review). In contrast, very little research has looked into the role of the feed-forward inhibitory pathway of the IN in normal and abnormal thalamocortical dynamics of health and disease, for example Crunelli et al. (1988), Zhu et al. (1999a) and Zhu et al. (1999b); these studies have emphasized both physiological and functional importance of the IN population in modulating LGN oscillatory activity. A similar trend is evident in computational model-based research on thalamocortical dynamics of health and disease that largely ignore the feed-forward inhibition by the IN; rather, the emphasis has been on the feedback inhibition by the TRN population. The interest in IN is revived in a recent set of experimental studies by Lörincz et al. (2009, 2008) emphasizing a vital role of the IN in modulating LGN oscillations in an awake cognitive state. In a previous work on a thalamo-cortico-thalamic model (Bhattacharya et al., 2011b), we have shown that the IN plays a role in “slowing” (left shift of the power spectral peak frequency) of alpha rhythms (8–13 Hz) recorded from the thalamo-cortical relay (TCR) cells and in the presence of cortical inputs, simulating an EEG biomarker of Alzheimer's disease. However, the non-triviality of the parameter space in the model puts a constraint on an in-depth understanding of the essential thalamic influence on cortical dynamics and vice-versa. Instead, we suggest that a “bottom-up” modeling approach, where the LGN cell dynamics corresponding to retinal input in both resting and awake conditions are studied independently of the cortical input, may better serve our goal of understanding the underlying cellular mechanisms of the IN circuitry and their role in the overall dynamics of the LGN. Indeed, the pioneering experimental studies that have provided the current fundamental knowledge about the thalamus were based on *in vitro* studies of LGN slices from mammals and rodents, when disconnected from the cortex. Here, based on insights from our prior model-based work, we follow the precedent set by the early experimental studies and model a de-corticated LGN responding to extrinsic input from the retinal pathway.

Several studies have shown that the LGN dynamics recorded from the TCR cells as LFP are correlated with an electroencephalogram (EEG) recorded from the occipital (visual) cortex. Thus, the time-series and power spectra from the TCR cells are considered as the “model output” and are validated qualitatively with reports of LFP (thalamus) and EEG (cortex) in existing literature. In Sections 3.1–3.4, the sensory input is taken as the background firing activity of the retinal ganglion cells in an eyes closed condition and is simulated with a “white” (flat power spectra) random noise. In Section 3.5, a case study is made to simulate Steady State Visually Evoked Potentials (SSVEP) in the model, a research paradigm that is increasing in popularity owing mainly to a direct correlation of the recorded brain response to a periodic input stimulus (see Norcia et al., 2015 for a review). Here, the model input is simulated with a periodic impulse train at a constant frequency in the range 5–50 Hz and superimposed on a white random noise, thus mimicking flashing LED lights provided as visual stimulus in SSVEP experimental studies.

The goal in this work has been to investigate the role of the afferent and efferent synaptic pathways of the IN population in effecting state transitions in the LGN circuitry. Toward this, systematic parametric variations with respect to their base values are explored in the model. The effect of synaptic depletion in the IN feed-forward pathway to the TCR is tested alongside the parametric variations, the objective being to draw a comparison with previous studies that ignore the IN population. Based on some preliminary results in Bond et al. (2014), our hypothesis in this work has been to understand possible underlying neuro-pathological changes that may “block” the IN inhibitory effects on the TCR cells, which may lead to state transitions in the LGN corresponding to both normal and pathological conditions. Indeed, experimental studies have shown the neuromodulatory effects of acetylcholine, noradrenaline (McCormick, 1992) and dopamine (Zhao et al., 2002) on the LGN that seem to “regulate” (under normal conditions) or affect (under abnormal pathological conditions) its synaptic efficacy and oscillatory patterns; specifically, dopamine has been implicated in affecting information transmission in the LGN by neuromodulation of IN. In this work, we have simulated the effects on neuromodulation by varying the neurotransmitter concentration in the synaptic clefts. However, for brevity in this study, we have maintained a single set of parameters for all neurotransmitter concentrations in the model (please refer to Equation 1); thus, any variation of parameter affects changes in all synaptic processes in the model.

The results validate several experimental reports and may be summarized thus: First, the IN population play a dominant role in efficient information transmission in the retino-geniculate pathway by suppressing the TRN feedback inhibitory effect on the TCR cells. Second, bifurcation to high amplitude synchronous oscillations are effected in the TCR cells with synaptic depletion and/or deletion in the feed-forward inhibitory afferent from the IN. A simultaneous decorrelation of the TCR output with retino-geniculate input is observed, thus simulating a transition from an awake state to a state of reduced cognition. Third, the IN plays a “homeostatic balancing” role in the LGN circuit, and its absence or reduced impact (owing to other related parametric deviation) may be speculated to aid unwanted periodic oscillations in the LGN that are often biomarkers of neuro-psychological disorders, for example “thalamocortical dysrhythmia” in schizophrenia or “slowing of alpha rhythms” in Alzheimer's disease. At the same time, a smooth transmission from alpha to theta band with a reduced inhibitory effect of IN simulates EEG of wake-sleep transitions. (The reader may please refer to Section 3 for detailed discussions, model validations and predictions).

The bidirectional connectivity between the thalamus and the cortex are well known to be fundamental to brain rhythms (Sherman, 2005; Destexhe, 2008). Specifically, the cortico-thalamic feedback is speculated to be a key factor in transition between a drowsy state showing theta rhythmic EEG to a deep sleep stage with delta rhythmic oscillations (Abeysuriya et al., 2014) and is consistent with studies on de-corticated thalamus Timofeev and Steriade (1996). Furthermore, physiological studies have attributed the delta band oscillations induced in the thalamus by cortico-thalamic inputs to a specific type of mGluR mediated synapse (Crunelli et al., 2006). However, the cortico-thalamic inputs are not modeled in this work; thus it is not surprising that there is a distinct lack of delta rhythms in the model output, the exception being the case corresponding to the total synaptic deletion in the self inhibitory pathway of the IN population. Our results have shown a non-linear relationship between the power spectral behavior of the model output and the IN self inhibitory mechanisms (the reader may please refer to Table 3, Figure 5); this non-linearity may imply a modulatory effect on the IN population. Along these lines, an experimental study (Lörincz et al., 2008) report that the impact of IN on thalamic oscillations could be identified only upon application of a cortico-thalamic stimulus. We hypothesize that the modulatory cortico-geniculate feedback to the IN targets its self inhibitory mechanism: a deletion of the pathway would lead to delta oscillations in the LGN. Ongoing work will extend our model by incorporating the thalamo-cortico-thalamic closed loop circuit to test this hypothesis. One other inconsistency in the model is that the GABA_*B*_ pathway does not seem to have a significant effect on the model output, and thus does not conform to experimental reports (von Krosigk et al., 1993). This calls for a further investigation of the parameters in this pathway, which is also expected to add to the dynamic repertoire of the model.

In conclusion, our model-based study implicates the IN population as a significant and vital constituent of the retino-geniculo-cortical pathway, regulating the state transitions of both TCR and TRN populations, and maintaining an overall homeostatic balance in the LGN circuitry in a normal awake state; any direct or indirect disruption to its synaptic mechanisms may cause unwanted brain rhythms that are EEG and LFP markers of neuro-psychiatric disorders.

## Author contributions

BB planned and designed the research and wrote the manuscript. TB and DT undertook parts of the technical simulation work during their 3rd year MEng project. LH co-supervised TB for the project work and introduced the concept of SSVEP in the model. SD provided consultation and guidance on validation of model results with EEG data and biological relevance.

### Conflict of interest statement

The authors declare that the research was conducted in the absence of any commercial or financial relationships that could be construed as a potential conflict of interest.
